# Distinct roles of *Hoxa2 *and *Krox20 *in the development of rhythmic neural networks controlling inspiratory depth, respiratory frequency, and jaw opening

**DOI:** 10.1186/1749-8104-2-19

**Published:** 2007-09-26

**Authors:** Fabrice Chatonnet, Ludovic J Wrobel, Valérie Mézières, Massimo Pasqualetti, Sébastien Ducret, Emmanuel Taillebourg, Patrick Charnay, Filippo M Rijli, Jean Champagnat

**Affiliations:** 1 NGI, UPR 2216, Institut de Neurobiologie Alfred Fessard IFR2218, Centre National de la Recherche Scientifique, F-91198 Gif sur Yvette Cedex, France; 2 IGFL UMR 5242 CNRS/INRA/UCB/École Normale Supérieure de Lyon, allée d'Italie, 69364 Lyon Cedex 07, France; 3 IGBMC, UMR 7104, CNRS/INSERM/ULP/Collège de France, CU de Strasbourg, F-67404 Illkirch Cedex, France; 4 Laboratori di Biologia Cellulare e dello Sviluppo, Università di Pisa, Via G Carducci, Pisa, Italy; 5 INSERM, U 784, Ecole Normale Supérieure, rue d'Ulm, 75230 Paris Cedex 05, France; 6 CEA, Laboratoire de Biochimie et Biophysique des Systèmes Intégrés, 38054 Grenoble, France

## Abstract

**Background:**

Little is known about the involvement of molecular determinants of segmental patterning of rhombomeres (r) in the development of rhythmic neural networks in the mouse hindbrain. Here, we compare the phenotypes of mice carrying targeted inactivations of *Hoxa2*, the only *Hox *gene expressed up to r2, and of *Krox20*, expressed in r3 and r5. We investigated the impact of such mutations on the neural circuits controlling jaw opening and breathing in newborn mice, compatible with Hoxa2-dependent trigeminal defects and direct regulation of *Hoxa2 *by Krox20 in r3.

**Results:**

We found that *Hoxa2 *mutants displayed an impaired oro-buccal reflex, similarly to *Krox20 *mutants. In contrast, while *Krox20 *is required for the development of the rhythm-promoting parafacial respiratory group (pFRG) modulating respiratory frequency,* Hoxa2 *inactivation did not affect neonatal breathing frequency. Instead, we found that *Hoxa2*^-/- ^but not *Krox20*^-/- ^mutation leads to the elimination of a transient control of the inspiratory amplitude normally occurring during the first hours following birth. Tracing of r2-specific progenies of *Hoxa2 *expressing cells indicated that the control of inspiratory activity resides in rostral pontine areas and required an intact r2-derived territory.

**Conclusion:**

Thus, inspiratory shaping and respiratory frequency are under the control of distinct *Hox*-dependent segmental cues in the mammalian brain. Moreover, these data point to the importance of rhombomere-specific genetic control in the development of modular neural networks in the mammalian hindbrain.

## Background

The role of hindbrain segmentation [1] in the organization and function of neural networks has been investigated using mutant mouse models for key regulatory genes, of which members of the *Hox *gene family are important. These genes display partially overlapping expression domains with rostral limits matching rhombomere (r) boundaries, providing a specific expression code for each segment along the anterior-posterior (AP) axis (reviewed in [1,2]). Segment-specific *Hox *expression is regulated by transcription factors exhibiting rhombomere-restricted expression patterns, such as Krox20 expressed in r3 and r5 [3-5], and by cross- and auto-regulatory activity of Hox proteins themselves [6-8]. Defining the biological significance of these rhombomere-specific gene regulatory networks is essential for understanding the development and functional organization of neuronal circuits in the vertebrate hindbrain. *Hoxa2 *is particularly interesting as it is the most anteriorly expressed *Hox *gene up to the r1/r2 border, and because it participates in complex rhombomere-specific regulatory pathways [6,9]. Targeted inactivation in the mouse revealed that *Hoxa2 *is indeed required for normal patterning of the rostralmost rhombomeres, as well as for the development of topographic brainstem circuitry [10-13]. However, the behavioral implication of *Hoxa2 *control has not yet been addressed.

A very sensitive method to evaluate behavioral significance of disturbed rhombomere development is to identify uncompensated abnormalities of vital postnatal behavior, for example, alimentary and breathing behaviors, *in vivo *in transgenic animals. The oro-facial control in particular is tightly linked with trigeminal sensory and motor pathways, as well as surrounding rhythmogenic and pre-motor reticular neurons [14-16]. Interestingly, mutations affecting rostral hindbrain segmentation differentially affect the control of jaw opening in neonates, which requires *Krox20 *[17] but not *Hoxa1 *expression [18]. A recent study [13] showed that *Hoxa2 *controls the connectivity pattern of the trigeminal sensory afferents to the rostral pons governing the formation of the whisker-to-barrel somatosensory circuit in the mouse. The implication of the *Hoxa2 *mutation on oro-buccal behavior remains to be investigated in these mice.

Breathing in rodents is thought to be governed by a rhythm generator named the pre-Bötzinger Complex (pre-BötC) [19,20], which acts as an oscillator. Previous studies from our group showed that it arises from post-otic rhombomeres [21,22]. The most recent findings support an additional parafacial respiratory group (pFRG) [23], which also shows an oscillating rhythmic activity. A dual origin of respiratory rhythm generation in newborn rodents involving a coupling between the pre-BötC and the pFRG [24] has been hypothesized and the roles of each oscillator in respiratory rhythm generation are still under discussion [25].

We have used the above-mentioned developmental approach to investigate the origin and the architecture of the respiratory rhythm generator. We previously described life-threatening anomalies of respiratory frequency that can be alleviated by naloxone in *Krox20*^-/- ^and *Hoxa1*^-/- ^mice. In these mice an anti-apneic system that exerts a rhythm-promoting function during the first postnatal days, likely the pFRG, is eliminated [17,18,26]. *Krox20*-dependent signaling and the r3-r4 segment are required in chicken and mice for the development of the pFRG [27].

In addition to the pre-BötC and the pFRG, the rostral pons has a role in control of breathing but its function in the intact animal remains questionable (see [28]). Distinct pontine inspiratory control *in vivo *has been recently proposed, the development of which can be altered by retinoic acid at embryonic day (E) 7.5 without affecting the respiratory frequency [29]. The present study investigates *in vivo *whether anti-apneic and inspiratory controls result from different AP specifications (*Krox20*-dependent, para-facial [26], and rostral [29], *Hoxa2*-dependent, respectively) caudal to the r2/r3 boundary. Alternatively, the present study also considers that abnormal inspiratory control *in vivo *may result from behavioral adaptation to r3-r5 defects in which *Krox20 *expression is altered but not entirely eliminated. These two hypothesis are investigated comparing *Krox20 *null mutant mice with null *Hoxa2 *[10] and hypomorphic *Krox20 *[30] mutant mice. We found that inactivation of either *Hoxa2 *or *Krox20 *impairs the rhythmic control of the jaw opening in agreement with HOXA2-dependent trigeminal defects and direct regulation of *Hoxa2 *by KROX20 in r3. However, an inspiratory pontine activity residing in the rostral pons and requiring an intact r2 is selectively abolished in *Hoxa2*^-/-^, but not in *Krox20*, mutants. These results indicate that pontine inspiratory and para-facial anti-apneic control systems are embryologically and functionally distinct and are under the control of distinct *Hox*-dependent segmental cues in the mammalian brain.

## Results

### Impairment of oro-buccal behavior in *Hoxa2*^-/- ^mice at birth

**Figure 1:**
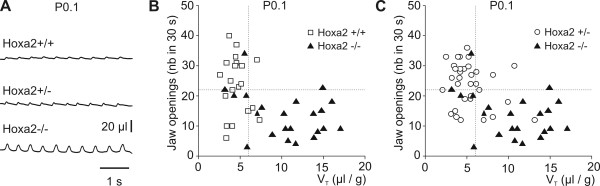
Phenotypic traits of *Hoxa2*^-/- ^mutants at birth: impaired oro-buccal behavior and increased tidal volume. **(a) **Plethsymographic recordings of wild-type (top), and heterozygous (middle) and homozygous (bottom) *Hoxa2 *mutant mice at P0. Inspiration is upward. Note that in *Hoxa2*^-/- ^mice, there is a two-fold increase in tidal volume compared with *Hoxa2*^+/- ^and wild-type littermates, whereas the frequency is the same (about 110 breaths/minute). **(b, c) **Individual data relating tidal volume (V_T_, abscissa) and number (nb) of jaw openings (ordinates) at P0.1. Each symbol corresponds to one animal. Black triangles are for *Hoxa2*^-/- ^mutants (b, c), open circles represent *Hoxa2*^+/- ^mutants (c) and open squares correspond to wild-type animals (b). Note that *Hoxa2*^-/- ^mutants can be separated from other genotypes at P0.1, due to their two-fold increased tidal volume and their reduced number of jaw openings. Broken lines indicate the values used to calculate penetrance of the phenotype (V_T_, all data inferior to mean – 1 standard deviation; jaw openings, all data superior to mean + 1 standard deviation).

To investigate oro-buccal behavior, we counted the number of jaw openings elicited by an oral stimulation in *Hoxa2*^-/- ^mice and compared the phenotype to that of *Krox20*^-/- ^mice. In *Krox20*^-/- ^mice, a reduction by about 50% of the number of jaw openings indicated an alteration of the trigeminal pre-motor and/or motor control, normally originating in the r2-r3 region [17]. In *Hoxa2*^-/- ^mutant mice, we found that the number of jaw openings during 30 s was decreased by 45% (*P *< 0.0001), ranging from 13.6 at postnatal day (P)0 to 16.6 at P0.5, significantly less than values of 24 at P0 and 32 at P0.5 measured in the wild-type (Table 1). Elicitation of jaw opening was normal in *Hoxa2*^+/- ^(Table 1) and *Krox20 *hypomorphic mutants (22 during 30 s (n = 7) versus 25 in the wild-type (n = 17)), indicating that full inactivation of either gene was required to affect behavior. The small number of evoked jaw openings at P0, coupled with the respiratory impairment described below, defined a highly penetrant (72%; 16 out of 22 homozygous mutants; Figure 1b, c) functional phenotype in *Hoxa2*^-/- ^mutant mice.

**Table 1:** Respiratory parameters and anatomical measures of wild-type, heterozygous and homozygous *Hoxa2 *mutant mice at birth

	Wild-type	Heterozygous	Homozygous
**Respiratory and behavioral parameters at P0.1**			
N	38	58	29
Birth weight (g)	1.45 ± 0.006	1.46 ± 0.022	1.40 ± 0.024
Weight gain at P0.75			
Grams	0.24 ± 0.01	0.22 ± 0.01	-0.09 ± 0.008*
Percent	16.5 ± 1.0	15.1 ± 0.9	-6.6 ± 0.6*
Jaw openings (in 30 s)	24.0 ± 0.41	23.9 ± 1.22	13.6 ± 1.63*
f_R _(cycles/minute)	107 ± 1.3	107 ± 4.0	110 ± 5.7
Tidal volume (μl/g)	4.6 ± 0.06	5.3 ± 0.41	10.6 ± 0.87*
V_E _(ml/g/minute)	0.52 ± 0.01	0.58 ± 0.06	1.21 ± 0.14*
Apneas (% of recording duration)	11 ± 0.8	9.6 ± 3.0	8.1 ± 3.7
**Anatomical measures**			
N	8	4	12
AP length of locus coeruleus (μm)	455 ± 18	420 ± 60	717 ± 23*
DV height of locus coeruleus (μm)	625 ± 60	700 ± 20	1,017 ± 73^†^
Distance between LC ventral limit (μm)	1,050 ± 25	Not measured	607 ± 79*

**Figure 2:**
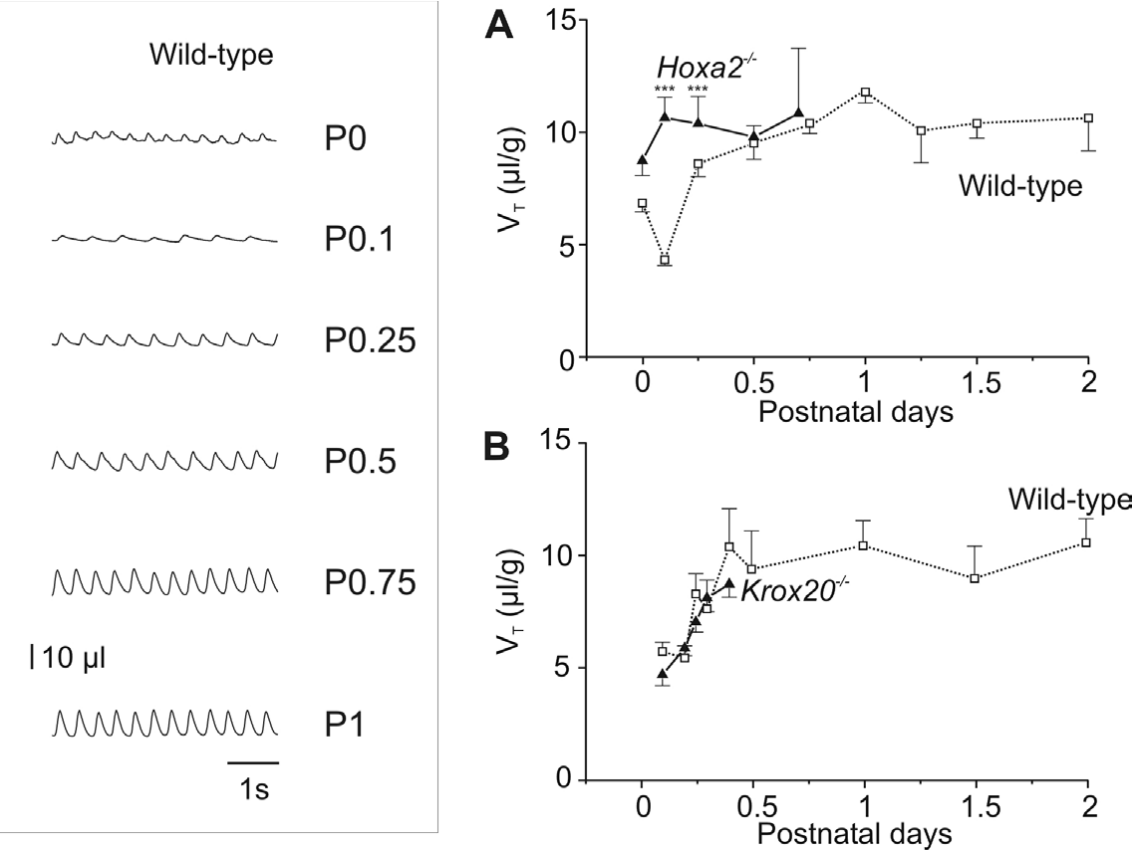
Tidal volume of *Hoxa2*^-/- ^and *Krox20*^-/- ^mutants during the first postnatal days. Inset on the left: plethysmographic recordings of wild-type mice at different times after birth (P0) during the first postnatal day. Inspiration is upward and expiration is downward. Note the evolution of tidal volume (respiratory amplitude) and respiratory frequency during the first day. Calibration bars: abscissa, 1 s; ordinates, 10 μl. Graphs present the evolution of mean ± standard error of the mean. Tidal volume (V_T_) in wild-type mice (open squares, dotted line) and **(a) ***Hoxa2*^-/- ^and **(b) ***Krox20*^-/- ^mutants (black triangles, continuous line) during the first two days after birth. All mutants were dead shortly after P0.75, therefore explaining the lack of further data. Note that in wild-type and *Krox20*^-/- ^animals, tidal volume rapidly increased during the first 12 hours of life whereas in *Hoxa2*^-/- ^mice, tidal volume was already two-fold greater at P0.1. ***P < 0.001.

Values are given as mean ± standard error of the mean. f_R_, respiratory frequency; V_E_, minute ventilation. Tidal volume at birth was increased two-fold in *Hoxa2*^-/- ^mice (see also Figure 2), but this feature disappeared rapidly: V_E _at P0.75 was not significantly different between the three genotypes. Note that *Hoxa2*^-/- ^mice lost weight between birth and P0.75, while their wild-type and heterozygous littermates gained weight. The birth weight and respiratory frequency are not significantly different. *P < 0.001; ^†^*P *< 0.01. DV, dorso-ventral; LC, locus coeruleus.

### Compared with the *Krox20 *null mutation, the *Hoxa2*^-/- ^mutation selectively affects the inspiratory amplitude without affecting respiratory frequency during the first postnatal hours

At P0.1, the tidal volume per body weight of *Hoxa2*^-/- ^homozygous mutants was twice as large as that of the wild-type or *Hoxa2*^+/- ^heterozygous mutant mice (Table 1). Samples of plethysmographic recordings (Figure 1a) show that this increase in respiratory amplitude (tidal volume (V_T_)) was not compensated for by any decrease in respiratory frequency, which was similar in the three genotypes at P0.1 (Table 1). Consequently, *Hoxa2*^-/- ^animals exhibited a greater than normal average respired volume (minute ventilation (V_E_) = f_R _× V_T_) at P0.1 (Table 1). Since there was no consistent difference in the duration of inspiration (Ti), these animals also showed a greater than normal inspiratory flow (V_T_/Ti). This was very different from the observations in the *Krox20 *null mutant mice, which showed a V_T _similar to their wild-type littermates (Figure 2b) during the first postnatal days.

In human infants, the first inspiratory efforts are the deepest breaths of the whole neonatal period [31-33]. The V_T _is later reduced and increases afterwards progressively during several hours (see page 26 of [34]). The same was observed in the wild-type (Figure 2a, b and inset) and *Krox20*^-/- ^(Figure 2b) mice, in which values of V_T _were reduced to about 5 μl/g at P0.1 and increased to about 10 μl/g at P0.5, which is the normal value maintained during the postnatal week (Figure 2a, b). At P0.5, the absolute tidal volume values of all genotypes were very similar, ranging from 8–11 μl/g. The tidal volume of *Hoxa2*^-/- ^mutant mice was the same as at birth, indicating that the decrease in V_T _observed in wild-type mice after the first breaths was impaired by the mutation (Figure 2a). Thus, the *Hoxa2 *invalidation perturbs a developmentally regulated mechanism involved in the control of the tidal volume during the first 12 postnatal hours.

**Figure 3:**
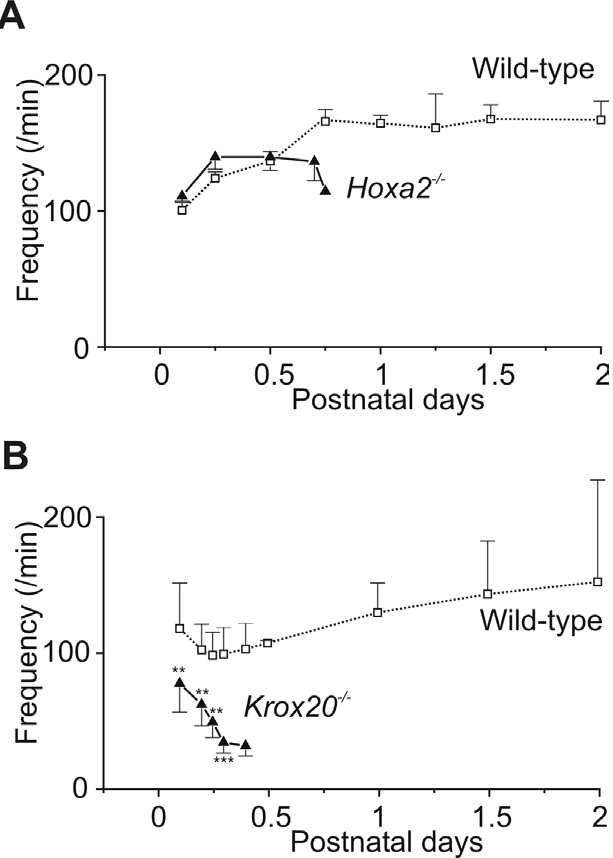
Respiratory frequency of *Hoxa2*^-/- ^and *Krox20*^-/- ^mutants during the first postnatal days. Graphs present the evolution of mean ± standard error of the mean respiratory frequency in wild-type mice (open squares, dotted line) and **(a) ***Hoxa2*^-/- ^and **(b) ***Krox20*^-/- ^mutants (black triangles, continuous line) during the first two days after birth. All mutants were dead shortly after P0.75, therefore explaining the lack of further data. Deficiency of the respiratory frequency is lethal in *Krox20*^-/- ^[17]; frequency is normal in *Hoxa2*^-/- ^mutants. **P < 0.01, ***P < 0.001.

Respiratory frequency (f_R_) also changed during the first day but increased only by about 50% (Figure 3a). The values of respiratory frequency at birth were about the same in *Hoxa2*^-/- ^mutants as in wild-type animals and both showed an increase in frequency between P0.1 and P0.5 (Table 1, Figure 3a). The changes of V_T _during the first hours were, therefore, mostly responsible for the modifications of the V_E _and both were abolished in *Hoxa2*^-/- ^mutant mice. This contrasted with the respiratory phenotype associated with the *Krox20 *null mutation resulting in a respiratory frequency 60% less than in the wild-type (42 ± 24 breaths/minute at P0.5, n = 17; Figure 3b). Very long apneas (> 3 s) were frequently observed and an increase in inspiratory flow did not compensate for the decreased frequency, thus minute ventilation at P0.5 (0.48 ± 0.38 ml/g/minute, n = 17) was less than half the wild-type values (1.10 ± 0.79 ml/g/minute, n = 25).

Thus, the respiratory phenotype of the *Krox20 *null mutation was dramatically distinct from that observed in *Hoxa2 *null mutants. *Hoxa2 *null mutation specifically affected the inspiratory control at birth, without modifying the respiratory frequency, whereas *Krox20 *null mutation had the exact opposite effect, deeply affecting the respiratory frequency without changing the respiratory amplitude.

### Partial impairment of *Krox20 *function does not mimic the *Hoxa2*^-/- ^respiratory phenotype and preserves breathing rhythm at birth

*Krox20 *is required for the development of r3, where *Hoxa2 *is expressed under Krox20 control [4]; nevertheless, the respiratory phenotype of the *Krox20 *null mutation was quite distinct from that observed in *Hoxa2 *null mutants (see above), suggesting that it was not induced by the loss of *Hoxa2 *expression in r3. However, as the *Krox20 *mutation results in the complete elimination of r3, it cannot be compared to the changes induced in the *Hoxa2 *null mutants, in which r3 is still present but displays patterning defects [10,11]. The abnormal inspiratory control in *Hoxa2 *mutants might, therefore, originate from an adaptive respiratory behavior following compensation for the loss of *Hoxa2 *in r3, while *Krox20 *is still functional.

**Figure 4:**
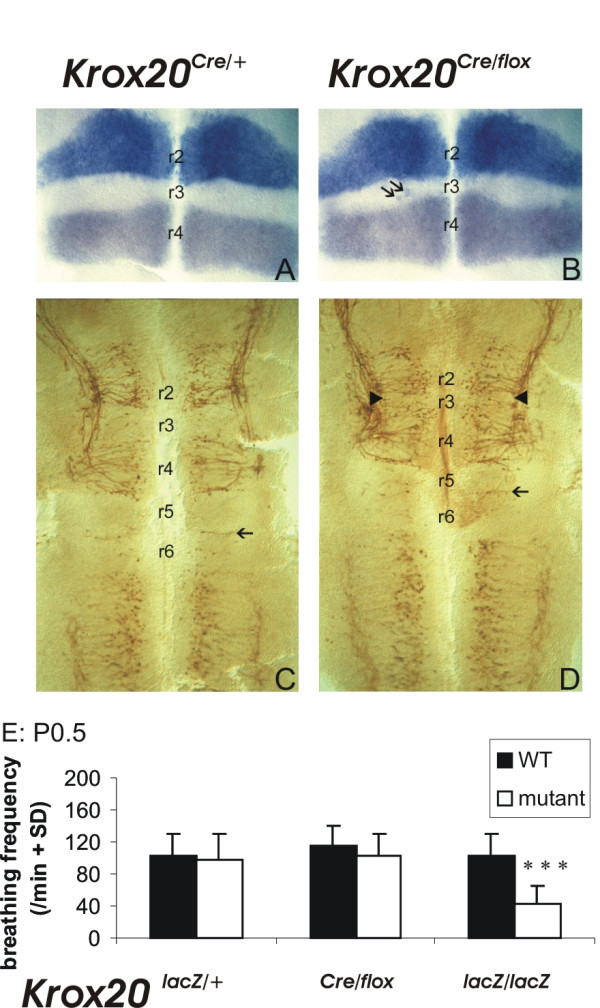
r3 and r5 are reduced in size in *Krox20*^*Cre*/*flox *^embryos. **(a, b) **The size of r3 was estimated at E9.5 on flat-mounted hindbrains by labeling adjacent rhombomeres. r4 was delimited by *in situ *hybridization with a *Hoxb1 *probe and r2 by detection of the alkaline phosphatase activity from an r2-specific transgene [47, 48]. The negative territory located in between corresponds to r3 and is reduced in *Krox20*^*Cre*/*flox *^(b) compared to control *Krox20*^*Cre*/+ ^(a) embryos. A few *Hoxb1*-positive cells are also observed within r3 in embryos (arrows). **(c, d) **Flat mounts of *Krox20*^*Cre*/+ ^(c) and *Krox20*^*Cre*/*flox*^(d) hindbrains immunolabeled with an antibody directed against the 155 kDa component of neurofilaments (2H3). r3 and r5 can be distinguished from even-numbered rhombomeres by their less advanced differentiation of reticular neurons, revealed by lower neurofilament immunoreactivity. The r5/r6 boundary is clearly visible since it is followed by axons (arrow in (c, d)). Both r3 and r5 are reduced in *Krox20*^*Cre*/*flox *^embryos, the effect being more dramatic in r3 (arrowheads). **(e) **Breathing frequency at birth in heterozygous *Krox20*^*lacZ*/+ ^(left) and in *Krox20*^*Cre*/*flox *^(middle) mice is the same as in wild-type mice (WT, white columns); it is lower than normal in homozygous *Krox20*^*lacZ*/*lacZ *^mice (right).  *** : p<0.001

To investigate this possibility we analyzed *Krox20*^*Cre*/*flox *^hypomorphic mutants resulting in a reduced, though not absent, r3 territory. This hypomorphic mutant was obtained by combining two previously developed *Krox20 *alleles, a Cre knock-in and a floxed allele (see Materials and methods for detailed description). Compound heterozygous *Krox20*^*Cre*/*flox *^mutants express *Krox20 *only transiently and, in the hindbrain, this results in a severe reduction of r3 (Figure 4). Analysis of the *Krox20*^*Cre*/*flox *^mutants revealed that the tidal volume was normal (115% of controls) as well as the respiratory frequency (P0.5: 101 ± 6 breaths/minute (n = 7) compared to 114 ± 10 breaths/minute (n = 17) in wild-type littermates; Figure 4) and the duration of apneas during the first postnatal day. Therefore, partial impairment of *Krox20 *function, resulting in a severe reduction of r3, is compatible with a normal control of the respiratory rhythm at birth and does not reproduce the *Hoxa2 *null phenotype characterized by the absence of a transient decrease in tidal volume around birth.

Because development of rhythmic circuits in r3r4 has been ascribed to r3-related control of neurogenesis in r4 [27], we analyzed the early pattern or neuronal differentiation in *Krox20Cre/flox *mutants (Figure 4c, d). We found that r3, although dramatically reduced in size, preserves its ability to delay neurogenesis and axonal invasion [35]. We conclude that function of the anti-apneic control does not depend on the ability to maintain quantitatively the size of embryonic territories, but rather as previously suggested [27], on odd-numbered rhombomere properties required to qualitatively control neuronal circuit formation.

Taken together, these results establish that Hoxa2 does not mediate the *Krox2*0 null respiratory phenotype in r3 since *Hoxa2 *null mutation does not reproduce *Krox20 *null mutation phenotypic traits. In addition, they suggest that the *Hoxa2 *mutant respiratory phenotype may be contributed by abnormalities in pontine areas more rostral than the *Krox20 *phenotype, since *Hoxa2 *has greater effects in its rostral domain of expression [10,36].

### Apneas are not responsible for the lethality of the *Hoxa2*^-/- ^mutation

At P0, the respiratory pattern was very irregular in *Hoxa2*^-/- ^mutant mice and the time spent in apneas (that is, respiratory pauses lasting more than 2 s) was about 10% of the total time of observation, similar to heterozygous and wild-type animals (Table 1). At P0.5, the time spent in apneas in wild-type animals normally decreased to 3.2 ± 2.0% of the recording time. In contrast, no decrease was observed in *Hoxa2*^-/- ^mutants (time spent in apneas at P0.5, 17.5 ± 5.5%) and at P0.5 these animals spent significantly more time in apneas than controls. However, apneas did not greatly influence the average volume inhaled by *Hoxa2*^-/- ^mutants. At P0.5, shortly before the death of the animals, the minute ventilation in homozygous mutants (1.36 ± 0.42 ml/g/minute) was not significantly different from that at birth (1.21 ± 0.14 ml/g/minute) or from that in the wild-type (1.71 ± 0.11 ml/g/minute) or heterozygous (1.43 ± 0.09 ml/g/minute) littermates.

**Figure 5:**
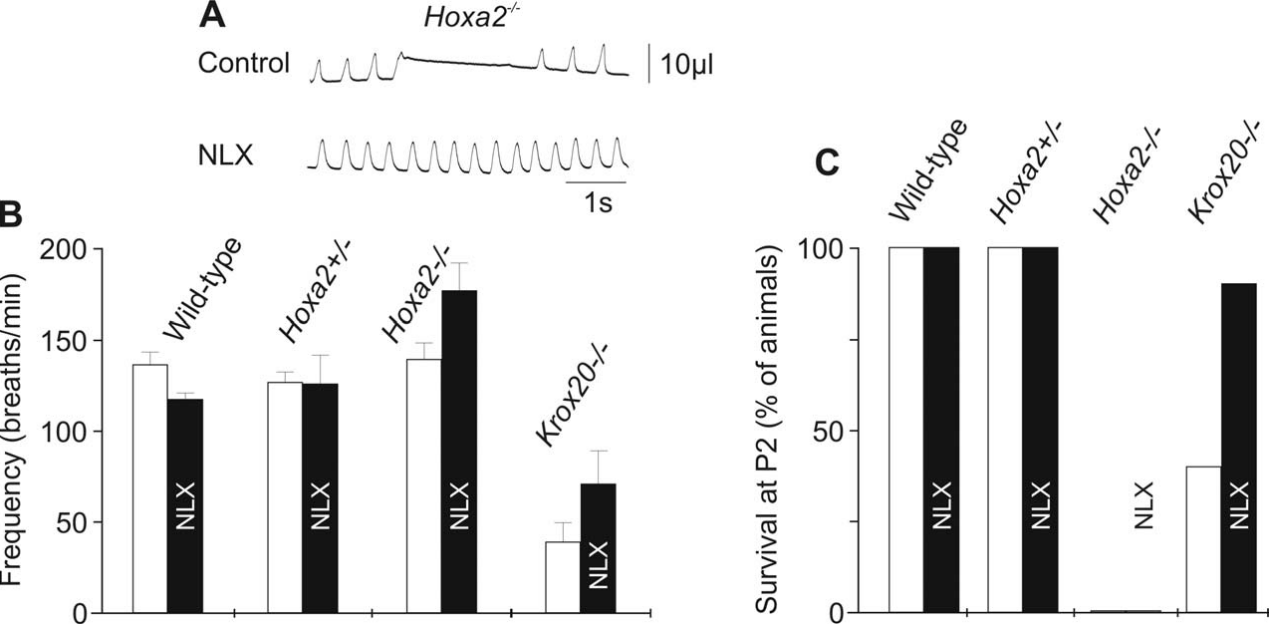
Naloxone treatment was effective on ventilation in *Hoxa2*^-/- ^mice but did not increase survival. **(a) **Plethysmographic recordings before (control) and after naloxone (NLX) treatment in *Hoxa2*^-/- ^animals. Note the frequency increase and the reduction of apneas. **(b, c) **Effects of the subcutanueous injection of NLX (1 mg/kg) at P0.5 upon mean ± standard error of the mean respiratory frequency calculated without apneic episodes in wild-type, *Hoxa2*^+/-^, *Hoxa2*^-/- ^and *Krox20*^-/- ^animals (b) or survival in the same genotypes (c). In (b, c) white bars indicate control values and black bars labeled NLX indicate values in the same animals one hour (b) (respiratory frequency) or 1.5 days (c) (survival) after NLX injection. Although NLX eliminates apneas (a) and increases respiratory frequency (by 31 + 11% in *Hoxa2*^-/- ^and 51 ± 37 % in *Krox20*^-/-^) (b), it does not allow survival of *Hoxa2*^-/- ^mutants (c).

Interestingly, administration of naloxone at P0.5 failed to improve survival of *Hoxa2*^-/- ^mutants (Figure 5), unlike *Krox20*^-/- ^and *Hoxa1*^-/- ^mutants [17,18]. In both wild-type and heterozygous *Hoxa2 *mutant animals, naloxone treatment had no significant effects on the breathing pattern. In the homozygous *Hoxa2*^-/- ^mutant mice (n = 7), naloxone injection at P0.5 slightly increased respiratory frequency from 135 breaths/minute to 177 breaths/minute (Figure 5b) and eliminated apneas (Figure 5a). Despite these stimulating effects upon ventilation, none of the treated *Hoxa2*^-/- ^mutants lived more than 18 hours (Figure 5c), the maximum lifetime observed in untreated *Hoxa2*^-/- ^animals. Altogether, these results demonstrate that apneas do not greatly influence the respiratory minute ventilation and are not responsible for lethality of the *Hoxa2 *mutation.

### Anatomical defects in rostral pontine areas in *Hoxa2*^-/- ^mutants

**Figure 6:**
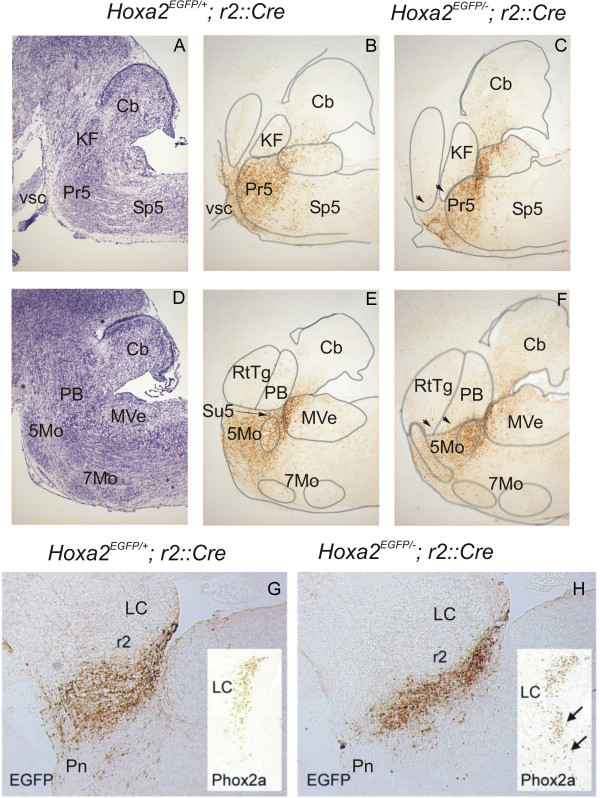
Hyperplasia of the r1-derived dorsal pontine tegmentum in *Hoxa2*^-/- ^mutants. Para-sagittal sections of the brainstem of **(a, b, d, e, g) ***Hoxa2*^*EGFP*(*lox*-*neo*-*lox*)/+^*;r2::Cre *(labeled *Hoxa2*^*EGFP*/+^*;r2::Cre*) or **(c, f, h) ***Hoxa2*^*EGFP*(*lox*-*neo*-*lox*)/-^*;r2::Cre *(labeled *Hoxa2*^*EGFP*/-^*;r2::Cre*) E16 mice at different latero-medial levels, from more lateral (KF level) **(a-c)**, to more medial (LC level) **(g, h) **through an intermediate level (trigemino-facial level) (d, e). **(a, d) **Violet cresyl stainings from which the delimitations of the different brainstem nuclei appearing as lines in other panels were drawn. **(b, c, e, f) **Immunodetection of EGFP, showing r2-derived cells. Note the reduction of the r2-derived domain and the ventral expansion of r1-derived nuclei such as the estimated KF (arrowheads in (c)), PB and RtTg (arrowheads in (f)) in *Hoxa2*^*EGFP*(*lox*-*neo*-*lox*)/-^*;r2::Cre *mice. **(g, h) **Immunodetection of EGFP at the LC level showing reduction of the r2-derived territory in *Hoxa2*^*EGFP*(*lox*-*neo*-*lox*)/-^*;r2::Cre*; insets show immunodetection of PHOX2A, a marker for the LC noradrenergic neurons – note the ventral expansion in *Hoxa2*^*EGFP*(*lox*-*neo*-*lox*)/-^*;r2::Cre *mice (arrows in (h)). 5Mo, trigeminal motor nucleus; 7Mo, facial motor nucleus; Cb, cerebellum; KF, estimated position of the Kölliker-Fuse nucleus; LC, locus coeruleus; MVe, medial vestibular nucleus; PB, estimated position of the parabrachial nucleus; Pn, pontine nuclei; Pr5, principal sensory trigeminal nucleus; RtTg, reticulo-tegmental nucleus of the pons; Sp5, spinal trigeminal tract; Su5, supratrigeminal nucleus; vsc, ventral spinocerebellar tract.

We analyzed the distribution of Enhanced Green Fluorescent Protein positive (EGFP+) cells in knock-in mice in which EGFP expression was selectively induced in r2-derived *Hoxa2 *expressing cells upon Cre-mediated recombination, by mating the *Hoxa2*^*EGFP*(*lox*-*neo*-*lox*) ^knock-in allele with a r2-specific Cre transgenic line [12] (Figure 6a, b, d, e, g; see also Materials and methods). This mating scheme resulted in full *Hoxa2 *targeted inactivation, and, concomitantly, allowed selective tracing of the *Hoxa2*-expressing EGFP^+ ^cells only in the r2-derived progeny.

Using heterozygous *Hoxa2*^*EGFP*(*lox*-*neo*-*lox*)/+^*;r2::Cre *animals at E18.5, we showed that the estimated lateral parabrachial medial and Kölliker-Fuse nuclei (Figure 6b, e), as well as the more medial noradrenergic locus coeruleus, identified by PHOX2A or tyrosine hydroxylase (Figure 6g), were located outside, though adjacent to, the trigeminal r2-derived domain. Therefore, the putative inspiratory related domain is derived from r1, about two rhombomeres distant from the r3-r4 region, the most anterior site known to induce formation of a respiratory frequency controller [17,26].

Analysis of EGFP expression in E18.5 *Hoxa2*^*EGFP*(*lox*-*neo*-*lox*)/-^*;r2::Cre *homozygous mutant mice (Figure 6c, f, h) indicated that the r2-derived peri-trigeminal nuclei were reduced while a ventral hyperplasia of the estimated Kölliker-Fuse (KF in Figure 6a–c) and parabrachial medial (PB in Figure 6d–f) nuclei was observed (arrowheads in Figures 5f and 6c). Other r1-derived structures, such as the locus coeruleus (LC in Figure 6c and Table 1) and the pediculo-pontine tegmental nuclei also expanded ventrally.

The anatomical structures modified in *Krox20*^-/-^*, Hoxa1*^-/- ^and *kreisler *mutants [17,18,29,37] have also been investigated in *Hoxa2*^-/- ^neonates. Specifically, we found a normal morphology of the anterior fourth ventricle, a normal AP length of the dorsal Pons, and a normal position of the ambiguus and facial branchial motor nuclei (data not shown). The parvocellular reticular nucleus, a dorsal pontine structure originating in r3 and extending between the trigeminal motor nucleus and the facial nerve [17,18,29] was also normal in *Hoxa2*^-/- ^mutants.

In conclusion, the brainstem defects likely responsible for the observed abnormalities of inspiratory control are restricted to the rostral pons of *Hoxa2*^-/- ^mutants and include the r1-r2 derived territory.

## Discussion

### The *Hoxa2 *mutation affects the development of the rostral pons

Consistent with the original observations of the *Hoxa2*^-/- ^defects being restricted to the anteriormost domain of *Hoxa2 *expression [10,36], the function of respiratory rhythm controllers located in the caudal pons and medulla was normal in *Hoxa2*^-/- ^animals. The phenotypic defects of the *Hoxa2*^-/- ^mice that were not observed in *Krox20 *mutants are likely to be related to the reorganization of neural structures derived from the alar plate at the r1-r2 level of the brainstem [10]. The distribution of cells expressing EGFP in the pons of *Hoxa2*^*EGFP*(*lox*-*neo*-*lox*)/-)^*;r2::Cre *homozygous mutants pons suggests that the territories derived from r2 are reduced along the AP axis or have lost r2-specific characteristics. Lack of an r1/r2 boundary [10-12] explains that territories derived from r1 and adjacent to r2 are also modified, with, for example, an ectopic projection of the ascending branch of the sensory trigeminal nerve to the cerebellum [13]. The abnormal location of locus coeruleus neurons is also consistent with an altered positioning of neurons within the r1-derived territory [11]. Thus, anatomical observations indicate that a large part of the rostral pontine territory deriving from r1-r2 is reorganized in *Hoxa2*^-/- ^mutants.

The r1-derived territory adjacent to r2 includes the estimated location of medial parabrachial and Kölliker-Fuse nuclei where a pontine respiratory group regulates adult mammalian inspirations [38-41] (for review see [28,42]). Therefore, *Hoxa2 *might be required for normal development of the pontine respiratory group. Our data further suggest that the pons contributes in shaping inspirations during the first postnatal hours in intact mice *in vivo*.

In addition, catecholaminergic [43] and cholinergic [44] control of breathing are probably affected. Interestingly, a large V_T _with normal respiratory frequency *in vivo *has been observed after the inactivation of the gene encoding acetylcholinesterase, a procedure found to greatly reduce the muscarinic and nicotinic control of the respiratory generator *in vitro *[44]. It is possible, therefore, that abnormalities of the pediculo-pontine tegmental nucleus (PPTg), the major source of ponto-bulbar cholinergic neurons, contribute to the control of inspirations in *Hoxa2 *mutants.

Selective inspiratory control by the rostral pons has been postulated to explain postnatal respiratory deficits following the exposure to sub-teratogenic doses of retinoic acid at the onset of hindbrain segmentation (E7.5) [29]. We presently show that development of inspiratory control requires rhombomere-related expression of the most rostral *Hox *gene in the neural tube. These results further suggest that the genetic network controlling hindbrain segmentation is involved in the formation of neuronal circuits controlling breathing, thereby giving the modular organization of the ponto-bulbar respiratory network [26].

### Control of the oro-buccal reflex requires both *Hoxa2 *and *Krox20*

As shown recently, *Hoxa2 *is required in r2-r3 for trigeminal nerve pathfinding at early stages and axonal arborization of afferents at later stages [13]. Here we show that the anomaly of facial circuits in *Hoxa2*^-/- ^mutant embryos is associated with an impairment of the reflex-induced rhythmic oro-buccal behavior at birth. Because the *Hoxa2*^-/- ^oro-buccal behavior resembles that induced by the elimination of r3 in *Krox20*^-/- ^[17], trigeminal behavioral deficits may involve both r2- and r3-derived processes, including the *Hoxa2*-dependent arborization of sensory axons in the rostral principal trigeminal nucleus [13]. In addition, *Hoxa2*^-/- ^and *Krox20*^-/- ^deficits may involve rhythmic pre-motor reticular neurons in the vicinity of the trigeminal motor nucleus controlling rhythmic opening of the jaw during alimentary behaviors [14-16,18]. This deficit in suction might participate in the early death of *Hoxa2 *null mutant mice, impeding the correct feeding behavior together with extensive transformation of the first branchial arch-derived facial skeleton [2,36].

### Robustness of *Krox20*-induced formation of anti-apneic circuits in the mouse

The most rostral rhombomere that has been shown to be required for the development of normal respiratory rate and to prevent apneas is r3. Elimination of *Krox20 *expression in r3 reduces quiet breathing frequency by 50% and multiplies by 10 the time spent in apnea, eventually leading to opioid-sensitive lethality [17,26]. Because, in contrast, hypomorphic *Krox20 *neonates breathe at a normal rate without apneas, *Krox20 *expression must be entirely eliminated to alter respiration. Our results, therefore, suggest that the transient expression of *Krox20 *and the subsequent reduction of r3 size observed in hypomorphic *Krox20 *mutants do not prevent the development of the para-facial anti-apneic system. This result extends to mice previous observations in chick embryos using loss- and gain-of function strategies [27]. *Krox20 *expression in r3 was found necessary for the non-cell autonomous induction of a neuronal rhythm controller from r4 [27]. In contrast, it was found to be unlikely that this induction requires the generation of a specific population of r3 neurons because KROX20 inhibits (rather than stimulates) neurogenesis and neuronal differentiation [35]. Furthermore, ectopic *Krox20*-expression in a limited territory (for example, obtained by unilateral electroporation in a rhombomere) was sufficient to induce a fully functional rhythm generator, although the population of neurons specified by *Krox20 *was certainly smaller than normal r3 in these preparations [27]. We presently show that, in mice as in chicks, induction of the anti-apneic parafacial function by *Krox20 *is a robust process that persists despite quantitative alterations of pontine cell populations. Our working hypothesis is, therefore, that *Krox20 *may act by initiating a cell non-autonomous control of specific neuronal fates rather than by generating a respiratory-related neuronal population in a cell autonomous manner. In addition, rhythm generators may compensate for minor abnormalities during fetal development to restore normal function at birth [29,44]. To further explore the parafacial development in mouse embryos, experiments are in progress in our laboratories to identify cell lineages that could be targets of the induction initiated by *Krox20 *expression.

### *Hoxa2 *is not a crucial target of *Krox20 *for the formation of the para-facial neuronal group controlling respiratory frequency

High levels of *Hoxa2 *are expressed in r3 and are required at late stages (E13) in trigeminal principal sensory neurons to induce arborization of whisker-related maxillary primary afferents [13]. In contrast, we show that *Hoxa2 *is not necessary in r3 for the development of the *Krox20*-dependent anti-apneic respiratory frequency controller; because in *Hoxa2*^-/- ^neonates respiratory frequency was normal, apneas were not life-threatening and treatment with naloxone had no effect on survival. In r3, *Hoxa2 *function downstream of *Krox20 *may be partially redundant with that of its paralogue *Hoxb2 *[11], also a direct target of *Krox20 *[3]. Functional redundancy of *Hox2 *paralogs can be expected at early developmental stages (end-segmental stages, about E9.5), during which *Krox20 *expression initiates formation of rhythm generators. Indeed, synergistic genetic interaction of *Hoxa2 *and *Hoxb2 *has been shown for the early patterning of r3 [11]. Redundancy might be less at later stages (about E13), when *Hoxa2 *exerts cell type-specific functions [13]. Previous observations [36] have shown that the anti-apneic activity abolished in *Krox20*^-/- ^and *Hoxa1*^-/- ^mutants is preserved in *kreisler *mutants lacking r5. Altogether, the available data support the location of the anti-apneic activity to be within the para-facial r3r4-derived territories and support *Krox20*, but not *Hoxa2*, as a major player in this process.

## Conclusion

We present evidence that distinct circuits regulate respiratory frequency and inspiration depth *in vivo *and involve different patterning mechanisms and progenitor populations during development. *Hoxa2 *inactivation, affecting the r1-r2 region and respiratory amplitude, did not severely perturb respiratory rhythm that requires normal *Krox20 *and *Hoxa1 *expression in the r3-r5 region [26]. In contrast, inactivation of either *Hoxa2 *or *Krox20 *impairs the rhythmic control of jaw opening, consistent with *Hoxa2 *being required for normal development of the trigeminal function [13] and *Krox20 *being a direct regulator of *Hoxa2 *in r3 [4,5].

## Materials and methods

All experiments were carried out following the ethical guidelines of the European Union Council (86/609/EU), the French Agriculture Ministry regulations for the care and use of laboratory animals in acute and chronic experiments. These experiments were also approved by the respective Institution Committees for animal care and handling.

### Mouse lines and genotype analysis

A total of 38 wild-type, 58 *Hoxa2*^+/- ^and 29 *Hoxa2*^-/- ^littermates resulting from crosses between heterozygous animals [10-12] were used in the present study. These numbers are in accordance with Mendelian repartition of genotypes (χ^2 ^test, *P *= 0.469). The *Hoxa2*^*EGFP*(*lox*-*neo*-*lox*) ^mouse knock-in allele allows selective activation of EGFP expression from the *Hoxa2 *locus only upon Cre recombinase mediated recombination, as described in [12]. The *r2::Cre *transgenic line allows selective expression of Cre in r2 and its derivatives, as described in [45]. The original *Hoxa2 *null mutation is described in [36]. The phenotypes of *Hoxa2*^-/- ^and *Hoxa2*^*EGFP*(*lox*-*neo*-*lox*)^/^*EGFP*(*lox*-*neo*-*lox*) ^homozygous mutants are indistinguishable. The analysis of *Hoxa2*^*EGFP*(*lox*-*neo*-*lox*)^/^+ ^and *Hoxa2*^*EGFP*(*lox*-*neo*-*lox*)^/^- ^specimens allowed, therefore, the comparison of heterozygous and homozygous *Hoxa2 *mutants carrying only one dose of *EGFP *in both genotypes. DNA was extracted from the tail of the neonate mouse and the genotype was subsequently determined by a PCR assay using specific sets of oligonucleotide primers, as described in [12]. All homozygous *Hoxa2*^-/- ^animals did not feed, lost 6.6% of their birth weight in their first 18 hours of life and died within 12–20 hours after birth, whereas their heterozygous or wild-type littermates fed, gained about 15% of their birth weight during the same period, survived (Table 1; see also [36,45]) and were, therefore, studied during the first week after birth.

We also analyzed 7 hypomorphic *Krox20 *mutants at P0.5 and P3-4 and we reinvestigated the breathing behavior of 17 *Krox20*^-/- ^animals [17] during the first hours following birth. The hypomorphic *Krox20 *mutant was obtained by combining two previously developed *Krox20 *alleles: the *Krox20*^*Cre *^allele consists of an insertion of the gene for the Cre recombinase into the *Krox20 *locus, resulting in *Krox20 *inactivation and expression of the Cre gene with a pattern that faithfully recapitulates the normal *Krox20 *pattern [46]; in the *Krox20*^*flox *^allele, the second *Krox20 *exon is flanked by loxP sites – this allele behaves like the wild-type until excision of the floxed exon by the Cre recombinase results in inactivation of *Krox20 *[30,46]. The compound heterozygous animals, *Krox20*^*Cre*/*flox*^, express *Krox20 *only transiently, due to subsequent elimination of the second exon. In the hindbrain, this combination behaves as a hypomorphic mutation, resulting in a severe reduction in, but not elimination of, r3, and a slight reduction of r5 (Figure 4).

### Plethysmographic recordings

Respiratory activity was measured using a modified barometric method, previously employed in neonates, called whole-body plethysmography [37]. The plethysmograph chamber (20 ml) equipped with a temperature sensor was connected to a reference chamber of the same volume. The pressure difference between the two chambers was measured with a differential pressure transducer connected to a sine wave carrier demodulator. The spirogram was stored on a computer using a Labmaster interface at a sampling frequency of 1 kHz. Calibrations were performed at the end of each recording session by injecting 2.5–5 μl of air in the chamber with a Hamilton syringe.

Measurements started 0.5–2 hours after birth and were repeated every 4–6 hours. Neonates were removed individually from the litter and placed in the plethysmograph chamber, which was kept hermetically closed and maintained at 31°C during the recording session (165 s). In each sample, periods of quiet breathing were identified by the absence of limb or body movements. Periods of limb, body and head movements were measured to determine neonatal activity during recordings. During quiet breathing, a computer-assisted method was used to measure the duration of inspiration and expiration from which respiratory frequency (f_R_) is derived and the tidal volume (V_T_, μl/g) from which minute ventilation (V_E _= f × V_T_/1,000, ml/g/minute) is derived. Total duration of apneas per recording session (given in percentages) provided an estimation of the apneic breathing. Apneas were included in the calculation of the minute ventilation, giving a mean value of this physiological parameter. In the case of naloxone treatment, separate calculations of respiratory frequency with and without inclusion of apneas were performed. Naloxone (1 mg/kg in saline) was administered subcutaneously using a Hamilton syringe, and the next recording carried out one hour after injection.

Before plethysmographic recordings, the number of jaw openings within 30 s was counted by introducing a catheter into the mouth of the newborns, therefore testing oro-buccal reflexes [17]. In order to obtain comparable data, counting was done by the same person for the whole study. Both plethysmographic recordings and jaw opening counting were done blindly, without knowing the genotype. Values are given as mean ± standard error of the mean. Comparisons between two sets of data were performed by paired or unpaired Student's T-tests.

### Anatomical observation and immunochemistry

E18.5 dpc (days post coitum) *Hoxa2*^*EGFP*(*lox*-*neo*-*lox*)/+^;r2-Cre and *Hoxa2*^*EGFP*(*lox*-*neo*-*lox*)/*EGFP*(*lox*-*neo*-*lox*)^;r2-Cre foetuses were obtained by caesarean section. Brains were dissected out, fixed in 4% paraformaldehyde, 1 × phosphate buffer saline (PBS) at 4°C overnight, cryoprotected, and sagittally sectioned. Adjacent sections were processed for immunohistochemistry using anti-EGFP (1:2000; Molecular Probes (Carlsbad, California, USA)) or anti-Phox2a (1:1,000; a gift from Jean-François Brunet, ENS, Paris, France) polyclonal antibodies. Sections were then incubated for 2 hours with biotinylated secondary antibodies (1:200, pH 7.4; Vector **(Burlingame, California, USA)**). Signal amplification was obtained by using the Vectastain ABC kit (Vector). Peroxidase was subsequently revealed in a staining mixture containing 0.05% 3,3'-diaminobenzidine hydrochloride (DAB, Sigma **(Lyon, France)**) and 0.03% H_2_O_2 _in 0.05 M TrisHCl pH 7.6.

## Competing interests

The author(s) declare that they have no competing interests.

## Authors' contributions

FC carried out the respiratory and oro-buccal behavior study of *Hoxa2 *null and *Krox20 *null mutant mice, started the anatomical study of *Hoxa2 *null mutants, participated in the conception and design of the study and drafted the manuscript. LJW and VM carried out the respiratory behavior of *Krox20 *hypomorph mutants. LJW and JC helped in the anatomical study of *Hoxa2 *null mice. MP and FMR generated the *Hoxa2*^*EGFP*(*lox*-*neo*-*lox*) ^and *r2::Cre *alleles, and performed the genotyping relative to these strains. MP and SD carried out the brain sectioning and EGFP staining of r2 specific Hoxa2-expressing cell progenies and FMR helped in the mutant analysis. ET and PC generated the two *Krox20 *alleles for the *Krox20 *null hypomorph mutant mice and did the genotyping relative to this strain. JC, FMR and PC conceived the study, and participated in its design and coordination and helped to draft the manuscript. All authors read and approved the final manuscript.
